# Es Colomer, a Unique Population of the Lilford’s Wall Lizard, *Podarcis lilfordi* (Squamata: Lacertidae)

**DOI:** 10.3390/ani15081093

**Published:** 2025-04-10

**Authors:** Ana Pérez-Cembranos, Valentín Pérez-Mellado

**Affiliations:** Department of Animal Biology, University of Salamanca, 37007 Salamanca, Spain; anapercem@usal.es

**Keywords:** Balearic Islands, insular lizards, body condition, lizard density, sex ratio

## Abstract

Es Colomer Island is occupied by a melanistic population of the Lilford’s Wall lizard, *Podarcis lilfordi*. Adult males are larger than females, with longer tails, and higher values of body mass, pileus length, head heigh, head width, hindleg length and number of dorsal scales. Adult sex ratio is balanced or slightly skewed towards males. At the summit of the islet, body condition was found to be significantly better than in almost vertical slopes. We found a higher proportion of digit amputations in adult males, probably due to frequent male–male interactions, promoted by high lizard density. During a 2024 survey, we recorded the highest known density of the Lilford’s Wall lizard. The prevalence of mites and blood parasites was 100%. The frequent intraspecific interactions, shown by the proportion of males with digit amputations, could be the source of the higher prevalence and susceptibility to blood parasite infections. The diet of lizards from Colomer is omnivorous, including several prey groups, as well as carcass remains from birds and mammals, and even conspecifics. Lizards from Colomer are an excellent example of the effects of extreme environmental conditions, with high population densities, strong intraspecific competition, the widespread presence of external and blood parasites, the extensive use of the scarce resources, and the opportunistic use of unpredictable resources in masting years, such as the fruits of the joint pine.

## Simple Summary

Colomer Island (northwest coast of Mallorca Island, Balearic Islands, Spain) is inhabited by an extraordinary population of the Lilford’s Wall lizard, *Podarcis lilfordi*, an endemic species of Balearic Islands that today is only present on the coastal islets of Mallorca, Menorca and Cabrera archipelago. The Colomer Wall lizards were discovered almost 100 years ago but have only been studied in the field since 2006. This is a population of melanistic lizards, with a marked sexual dimorphism in several characteristics such as body size, tail length and head size. The density of lizards is very high. In 2024 we recorded the highest known density of lizards in this species. This remarkable abundance promotes the existence of frequent aggressive interactions between males, a high parasite load and a foraging ecology that includes a large variety of prey, plant matter, carcasses from birds and mammals, and even conspecifics. All these traits are considered an adaptation to the extreme environmental conditions of this small Mediterranean islet.

## 1. Introduction

**Figure 1:**
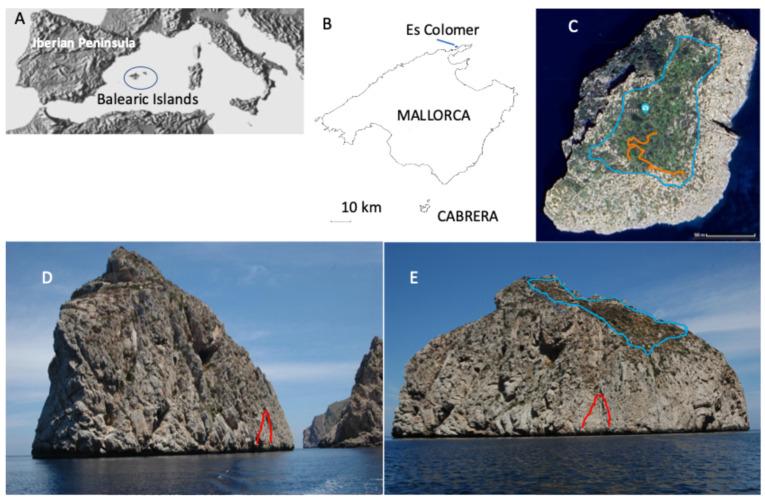
(**A**) The location of the Balearic Islands (Spain) in the Western Mediterranean Basin. (**B**) Situation of Colomer Island on the Northwestern coast of Mallorca Island. (**C**) Aerial view of Colomer Island. The blue line delimits the summit area sampled in 2008, 2022 and 2024. The orange line indicates the approximate paths of the line transects carried out in 2024. (**D**) Southern slopes of Es Colomer Island. On the right, the red line indicates the lower area sampled in 2006, during the first visit to the island. (**E**) View of Es Colomer Island from the east. The sampling area from 2006 is indicated (red line) along with the upper area studied in 2008, 2022 and 2024 (blue line).

The population of the Lilford’s Wall Lizard, *Podarcis lilfordi* (Günther, 1874) on Colomer Island (Figure 1) was first mentioned by George A. Boulenger in his seminal monograph on lacertid lizards [1], where he gave body measurements and scalation counts of three adult males collected by M.G. de Southoff in 1917. Apparently, the same collector returned to Colomer in 1928, obtaining 33 additional specimens that were the base for the description of the subspecies *Podarcis lilfordi colomi* by Salvador [2]. This author published body measurements and scalation data from an unknown number of specimens, without any statistical analysis (see also, [3,4]). Before Salvador [2], the paleontologist Guillem Colom published a note on this population, describing some specimens obtained from a local fisherman [5].

**Figure 2:**
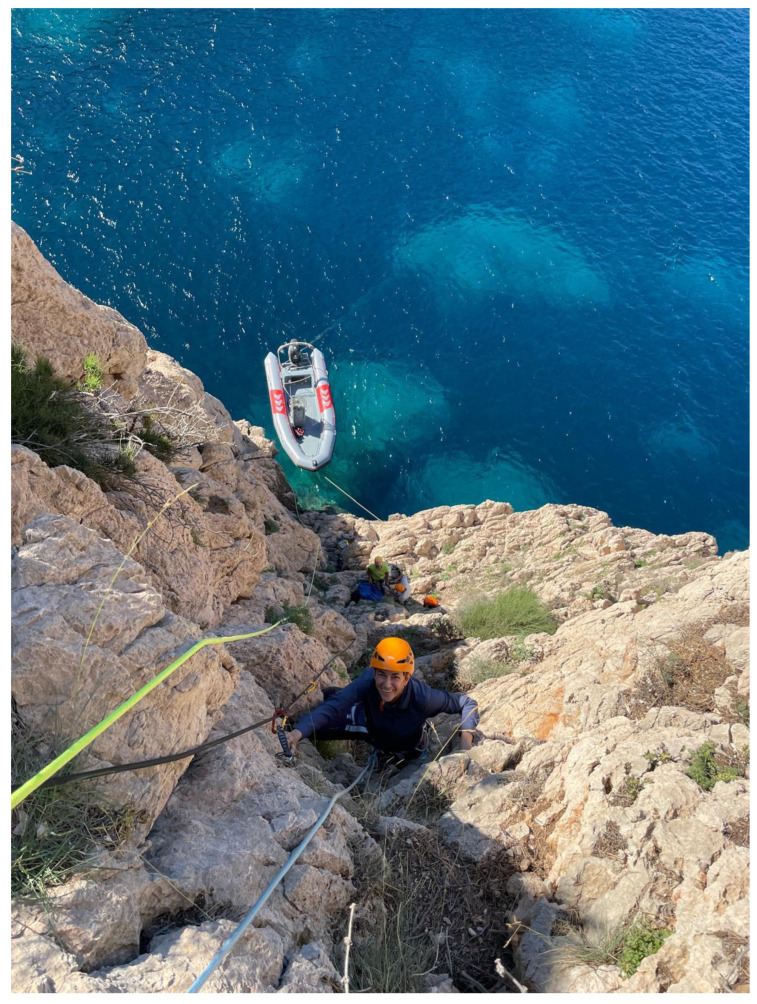
Access to the islet is only possible by means of classic climbing equipment from a boat. In the almost vertical climbing area (slope area), there is poor plant cover, but a constant presence of lizards.

Although this population has been known about for more than one hundred years, it has barely been studied, probably because of the difficulty of access to the islet. It was the last coastal islet of the Balearic Islands to have its flora studied [6]. Disembarking at and climbing the eastern slope of the islet, the only accessible one, is somewhat difficult (Figure 2), which has allowed this unique population to be preserved in a good conservation state. It is however surprising that, during an unknown period in the 20th century, the islet was apparently home to a small group of introduced sheep [7,8]. Fortunately, today, this invasive species is absent.

Pérez-Mellado et al. [9] considered that a line transect estimation of lizard density was impractical at Colomer. However, in later visits to the islet, we were able to reach summit areas where line transects were possible (Figure 1).

In a previous work, we proposed that the population of Colomer could be the best representation of very large extinct population of the Balearic lizard from Mallorca Island. During the Holocene, around 2000 years ago, the species became extinct on the main islands of Mallorca and Menorca, probably because of the introduction of terrestrial predators by the Romans [10]. It now survives only on the small islets found around the coasts of Menorca, Mallorca and Cabrera Archipelago. The three haplotypes described by Colomer are separated from other clades of *P. lilfordi* by a minimum of 10 mutational steps [11,12,13]. From a molecular viewpoint, Colomer arranged them within Clade C, together with northern populations of Cabrera Archipelago. According to these results, we propose that the Cabrera Archipelago acted as a refuge area, within which Mallorca was later recolonized [11]. Then, outlier SNPs revealed a higher degree of similarity between Colomer and Dragonera lizards. This result, together with the high migration rate between Mallorca Island and Cabrera, supports the proposal that Colomer Island is the home of a relict population representative of the early population that once colonized Mallorca Island [14].

In this study, we describe the morphometric traits of Colomer lizards that are not provided in the original description of the subspecies, as well as lizard density, trophic ecology and parasite load. Our objective is to update our knowledge of this unique population, giving useful information related to conservation planning for this population.

## 2. Materials and Methods

### 2.1. Study Area

**Figure 3:**
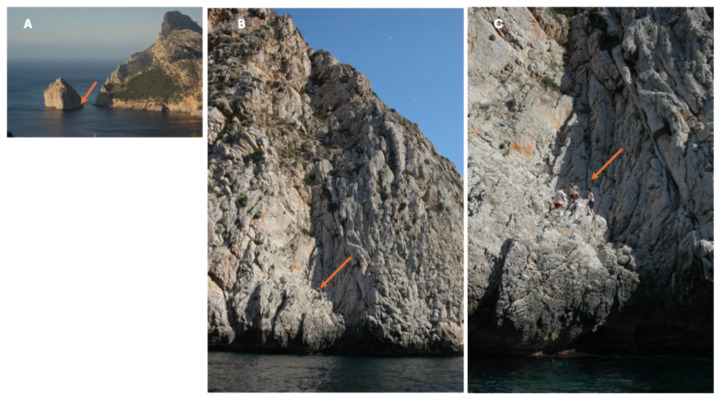
(**A**) A view of Colomer Island from the panoramic viewpoint on Mallorca Island, from which the island has been photographed thousands of times by tourists. (**B**,**C**) The area where field work was performed in 2006 is indicated by the orange arrows.

Colomer is one of the most photographed islets of the Balearic Islands [8] (Figure 3). It is a limestone islet (39°56′42″ N, 3°71′53″ E, Pollença, Mallorca, Balearic Islands, Spain), that is very isolated near the northwestern coast of Mallorca Island. Its surface has been described as ranging from 2.75 to 3.05 hectares, depending on the source, with only around 2.5 hectares covered by plants (Figure 3). The flora of the islet are relatively rich, with 139 vascular plants recorded [6], of which at least 15 are endemic to the Balearics [8]. The most important shrub, from the point of view of lizard use, is the joint pine, *Ephedra fragilis*. However, there are abundant patches of *Limonium balearicum*, *Crithmum maritimum*, *Daucus carota*, *Asparagus* spp. and *Atriplex prostata*. Ground cover was visually determined [15] as a soil surface covered with plants, rocks or gravel. In 2008, we measured cover in terms of intercepts of plants and open areas along a line of 25 m length. At the summit area of the island (Figure 1C,E), we randomly performed 10 transects of 25 m. Shrubs occupy the majority of the surface (58.04%) with a predominance of *Asparagus* spp. (21.91%) and *Ephedra fragilis* (15.82%).

We observed some breeding pairs of the Eleonora’s falcon, *Falco eleonorae*, as well as a large breeding colony of the yellow-legged gull, *Larus michahellis*, and some terrestrial birds. *Columba livia*, *Falco peregrinus*, *Monticola solitarius*, *Sylvia balearica* and *Apus pallidus* were also recorded on the island [6]. It is also interesting to note the presence of some endemic snails (terrestrial Gastropoda) such as *Iberellus balearicus*, *Tudorella ferruginea* and *Xeroplexa frater*. Even though the islet is very rarely visited, we detected the presence of ship rats, *Rattus rattus*, close to the shore, and even at the summit of the islet.

### 2.2. Study Period

The islet was visited on four occasions—2006, 2008, 2022 and 2024. During the visits of 2008, 2022 and 2024, we were able to reach the summit of the islet (Figure 1C,E). We visited the islet for day-long periods, staying around 4–5 h during each visit. In 2006, during the first visit, we only worked on the lower eastern slopes, closer to the shore (Figure 1D and Figure 3). We have compared the results of our analyses between two locations, slope and summit, and two seasons, spring and summer.

### 2.3. Lizards

*Podarcis lilfordi* (Günther, 1874) (Squamata, Lacertidae) is a medium-sized lizard with a maximum snout–vent length of 81 mm in males and 75 mm in females. It is an endemic species inhabiting the coastal islets of Menorca, Mallorca, and Cabrera Archipelago [4]. The Lilford’s Wall Lizard is an active forager that captures insects and other invertebrates, but it also consumes vegetal matter, carrion, and conspecifics [16,17].

We studied in the field 52 individuals that we captured and then released during our visits to the island. In addition, we studied 36 lizards that had been deposited in the Natural History Museum of London by M.G. de Southoff between 1917 and 1928, including type specimens of the subspecies *Podarcis lilfordi colomi* described by Salvador [2]. During three visits (2006, 2008 and 2022), lizards were noosed, studied in place, and then released at the site of capture. The lizards were active even from few meters above the shore (Figure 3). However, the bulk of the population lives around the summit, in areas of denser and more diverse vegetation cover (Figure 1D,E).

### 2.4. Morphological Analysis, Body Condition, and Sex-Ratio

The morphology of the lizards was studied, including six body dimensions, as well as body mass (weight). We measured snout–vent length (SVL), intact tail length (TL), pileus length (PL), head width (HW), head height (HH) and hindleg length (HLL). All measurements were taken with a digital calliper to the nearest 0.01 mm, apart from SVL and tail length, which were measured with a steel rule to the nearest 1 mm. Weight was obtained with a spring scale Pesola® with a precision of ±0.25 g. In all lizards we registered if the tail was intact or regenerated. The presence of missing toes was recorded in museum specimens and only in captured lizards at the summit zone in 2022. Six scalation characteristics were recorded—gularia, collaria, dorsalia, ventralia, left femoralia, and left fourth digit lamellae (see [18] for methodological details of body measurements and scalation counts). Not all characters were recorded in all individuals. The log-transformed values of morphometric and scalation characteristics were compared between adult males and females using one-way ANOVA for SVL and ANCOVA analyses for the remaining characters, using SVL as a covariate. The minimal model was finally tested with ANOVA [19].

We employed the Scaled Mass Index (SMI) as a body condition estimation. This index offers an accurate method to adjust variables measured with different scales [20]. Sex ratio was calculated as Adult Sex Ratio (ASR) in captured lizards and observed lizards during line transects. ASRs were tested with the binomial test [19]. A smaller sample of adult lizards from Colomer was included in a previous morphometric work on *P. lilfordi* [21].

### 2.5. Mites and Blood Parasites

Mites were counted in the field with a 5× magnifying lens, summing the total numbers of mites found in the head, gular, ventral, pericloacal, dorsal, and tail regions.

To obtain blood samples, we made a slight cut in the dorsal side of the tail with a sterile scalpel. With the detached blood drop we obtained a blood smear. Also, some blood samples were obtained by clipping off the tail tip, using tail tips to extract DNA for genetic studies [11,12]. Blood smears were placed on microscopic slides and air-dried in the field. In the lab, slides were fixed with absolute methanol for 10 min and then stained with modified Giemsa for 20 min. The samples were analyzed using an optical microscope at 400×. The only blood parasites identified with microscopic inspection were hemogregarines [22,23]. The intensity of parasitism was estimated as the number of infected cells on a total of 2000 cells per sample. Prevalence was estimated as the percentage of infected individuals at each area (slope and summit) of Colomer Island. We obtained data on blood parasites from 34 adult lizards (24 from the slope in 2006 and 2008 and 10 from the summit in 2008).

### 2.6. Lizard Abundance

Abundance estimates of lizards have been carried out with line transects. The reliability of the line transect is comparable to that of other density estimation methods if it is applied rigorously, respecting the premises of the estimation models and by observers with enough experience [24,25] (but see also [26]). In 2022, line transects were carried out in the last section of the upload hillside of the island, in an area of very strong slope and poor plant cover. Line transects made in 2008 and 2024 covered a larger extension in the summit area of the island (Figure 1C,E).

The density calculations were carried out with the ‘unmarked’ package in the R environment [27,28]. This package works with N-mixture models that allow precise estimates of abundance in small vertebrates [25]. To build the abundance model, we used the “distsamp” function that fits a multinomial Poisson model to the distance data [29]. The probability of detection in the density estimation was modeled as a function of the perpendicular distance (d) to the observer, using the “half-normal” detection function of unmarked [27]. We used the detection probability g(x) along a two-meter-wide band on both sides of the transect line. For each estimate, we present the density as the number of individuals per hectare ± the standard error (SE). We also include the number of contacts and the length of the transect in meters. In all transects, we include adult individuals of both sexes together and a very small fraction of individuals that, due to their body size, would be classified as juveniles or subadults. This juvenile fraction is extremely small in most transects.

### 2.7. Diet

Scats were obtained directly from the ground or from captured lizards that defecated during handling. During visits to the island, 236 fecal samples were obtained, both from the eastern slope of the islet and from the summit. We comparatively analyzed the diet in spring (May) and in summer (June and August). The comparison between the slope and summit areas was carried out only with the 198 fecal samples corresponding to 2006, 2008 and 2022, since in 2024, the samples from both areas were not separated during the field work. We analyzed fecal samples under a binocular dissecting microscope. In lizards, diet reconstruction based on a meticulous fecal pellet analysis has been found to be highly comparable to diet reconstructions based on gastric contents removed from dissected stomachs, with soft-bodied prey and particularly insect larvae being equally represented in fecal pellets and gut contents [30]. Each individual scat was spread in a thin layer of less than 0.5 mm over the entire surface of a Petri dish with some drops of 70° ethylic alcohol. The percentage of vegetal matter was then visually estimated according to the surface occupied by vegetal remains. Prey remains were identified up to the order or, rarely, family level. Prey number for each fecal pellet was conservatively estimated by counting only easily identifiable remains. The consumption of carcasses from birds and mammals was inferred from the presence of individual fragments of feathers or hairs, employing the work of Teerink [31] for hair identification.

We calculated prey abundance (%n) as the percentage of a given prey type in relation to total prey number, and the relative prey or plant presence (%p) as the percentage of feces containing a given prey type or plant. The diets of lizards from spring and summer and from the slope and summit areas were compared with a permutational multivariate analysis of variance (permutational MANOVA) via the ‘adonis’ function from the ‘vegan’ R package [32], using the area (slope and sumit) and the season (spring and summer) as explanatory variables and including in the model the interaction between area and season. The multivariate homogeneity of group dispersions (variances) was tested with the function ‘betadisper’ from the ‘vegan’ package.

We estimated and compared diet diversities via the approach proposed by Pallmann et al. [33]. Instead of describing diet diversity through a given index, we converted these “raw” indices into “true” diversities, which all belong to one and the same mathematical family. That is, different measures are considered special cases of Hill’s general definition of diversity measure [34]. In this way, to study differences in diversity among diets corresponding to different seasons and zones, we performed two-tailed tests for integral Hill numbers. This selection included the transformed versions of the three following indices: the species richness index, Hsr (q = 0); the Shannon entropy index, Hsh (q → 1); and the Simpson concentration index, His (q = 2 [35]). We performed 5000 bootstrap replications to obtain reliable *p*-values. The methods described here are implemented in the R package ‘simboot’ [36].

## 3. Results

### 3.1. Morphometry, Sexual Dimorphism, and Sex-Ratio

**Figure 4:**
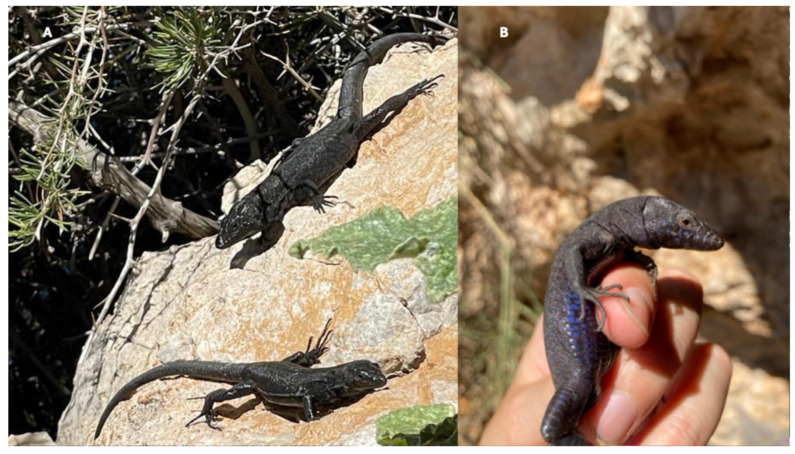
(**A**) A couple of basking *Podarcis lilfordi colomi,* an adult male above and an adult female below. (**B**) Cobalt blue spots of an adult male, arranged in two longitudinal series on each side of the ventral zone.

We studied 88 lizards, including the holotype and two paratypes of the subspecies *P. lilfordi colomi* [2]. In Table 1, we summarize the morphometry of adult lizards. Males are significantly larger than females (Table 1; Figure 4). In addition, the population showed a significant sexual dimorphism, with longer lengths of intact tails, larger body masses, and higher relative values of pileus length (PL), head height (HH), head width (HW), hindleg length (HLL), and the number of dorsal scales (Dorsalia) in males. The number of ventral scales (Ventralia) was found to be significantly higher in females (Table 1).

From captured lizards, sex ratio (males/females) was balanced both in the slope area (10/15, binomial test, *p* = 0.424) and at the summit of the islet (13/12, *p* = 1). If we consider the adult sex ratio (ASR) observed during line transects at the summit, there is weak evidence against a 50:50 sex ratio (57/37, binomial test, *p* = 0.049). Males and females from slopes are similar in body size (SVL) to lizards from the summit of the islet (males, one-way ANOVA, F_1,20_ = 3.282, *p* = 0.085; females, one-way ANOVA, F_1,25_ = 1.11, *p* = 0.302). However, the body condition of adult lizards was significantly better at the summit of the islet (one-way ANOVA F_1,32_ = 16.23, *p* = 0.0003; summit$—\bar{x}$ = 10.79 ± 0.45, range—8.93–13.47, n = 10; slope$—\bar{x}$ = 8.93 ± 0.23, range—6.78–10.99, n = 24).

Digit amputations were significantly more frequent in adult males (65.71%, n = 35) than in females (27.27%, n = 11; Fisher exact test, *p* = 0.009), while the proportion of adult males with regenerated tails (80.85%, n = 47) was statistically similar to that for females (74.19%, n = 31; Fisher exact test, *p* = 0.79).

### 3.2. Parasite Load

The prevalence of mites was 100%. The intensity of ectoparasite load was similar in adult males and females (F_1,23_ = 0.002, *p* = 0.961). Thus, we have pooled the results of both sexes in the next analyses. We did not find any correlation between the intensity of ectoparasite load and the body size (SVL) of lizards (Spearman rank correlation, S = 1828.8, σ = 0.296, *p* = 0.149). Ectoparasites were significantly less frequent in lizards found at the summit (one-way ANOVA, F_1,23_ = 15.34, *p* = 0.0006; summit—$\bar{x}$ = 45.9 ± 9.93, range—9–104, n = 10; slope$—\bar{x}$ = 212.67 ± 33.9, range—24–571, n = 15).

The prevalence of infection by hemogregarines was also 100%. In the slope area, we did not find differences in the intensity of blood parasitism in the two sampled years (one-way ANOVA, F_1,23_ = 0.001, *p* = 0.977; May 2008, $\bar{x}$ = 36.93 ± 11.56, range—2–167, n = 14; June 2006, $\bar{x}$ = 36.3 ± 20.7, range—1–218, n = 10). In addition, the intensity of blood parasitism was similar in males and females (F_1,32_ = 1.573, *p* = 0.219). The intensity of blood parasitism was significantly higher in the summit area (one-way ANOVA, F_1,32_ = 5.808, *p* = 0.021; summit$—\bar{x}$ = 92.4 ± 23.48, range—10–203, n = 10; slope$—\bar{x}$ = 36.93 ± 11.56, range: 2–167, n = 24). According to these results, there is a negative and significant correlation between ectoparasite load and the intensity of blood parasites in individual lizards (Spearman rank correlation, S= 9903, σ = −0.5131, *p* = 0.001).

### 3.3. Abundance

Lizard density was estimated on the slope during our 2022 survey and at the summit in 2008 and 2024 (Table 2), with a variable number of contacts. In June 2024, we recorded a very high lizard density in the summit area, with more than 5000 lizards per hectare (Table 2).

### 3.4. Diet

**Figure 5:**
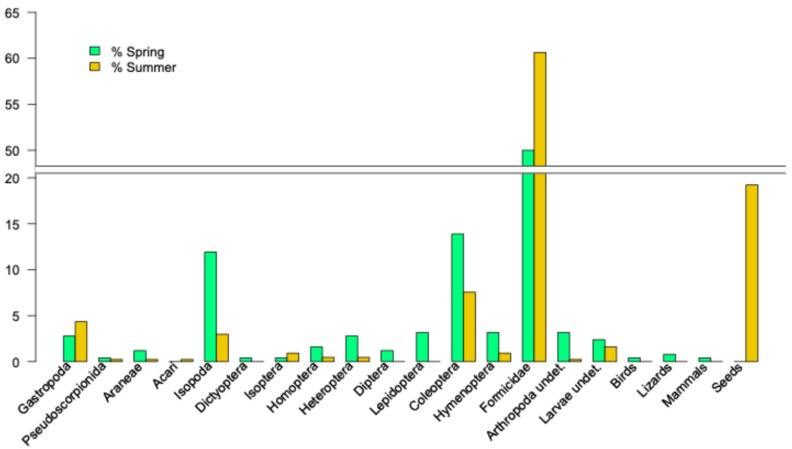
Spring and summer diets of *Podarcis lilfordi* in Colomer Island. Percentages of different prey types.

From the analysis of 208 fecal samples, we identified 689 prey items. The diet of lizards from Colomer is based on small arthropods, mainly ants, beetles, and isopods (Table 3). During summer, there is a notable contribution is made by seeds, particularly from *Ephedra fragilis*. The permutational MANOVA results indicate that the diets were similar in slope and summit zones (F_1,197_ = 3.3905, *p* = 0.052), but significantly different between spring and summer (F_1,197_ = 11.4195, *p* = 0.001; Table 3 and Figure 5), without any interaction between zone and season factors (F_1,197_ = 3.7089, *p* = 0.06). The homogeneity of the dispersion test confirms that the significant differences between spring and summer are not due to unequally dispersed distributions (F_1,196_ = 3.7293, *p* = 0.054).

The volume of plant matter was also significantly higher during spring (one-way ANOVA, F_1,196_ = 4.085, *p* = 0.04; spring$—\bar{x}$ = 55.83 ± 3.68, range—0–100, n = 134; summer—$\bar{x}$ = 42.77 ± 5.3, range—0–100, n = 64). However, during June 2024, a masting period of *Ephedra fragilis*, 91% of feces samples contained seeds of *E. fragilis*. The levels of consumption of plant matter were similar in the slope and summit zones (one-way ANOVA, F_1,196_ = 1.364, *p* = 0.244; slope$—\bar{x}$ = 45.23 ± 6.41, range—0–100, n = 47; summit$—\bar{x}$ = 53.59 ± 3.46, range—0–100, n = 151).

Diet diversity was higher in spring (Simpson index = 0.7528 ± 0.0005) than in summer (Simpson index = 0.4339 ± 0.0011), but we only found significant differences (*p* = 0.035) in Hill’s number q = 0, corresponding to species richness index, H_sr_, which strongly emphasizes rare species by weighting all species equivalently and irrespective of their frequency of occurrence [33]. During summer, some prey items, such as Lepidoptera, Diptera, Dictyoptera, or carcasses of birds and mammals, disappeared, and the diets were dominated by the consumption of Formicidae and seeds of *Ephedra fragilis* (Table 3 and Figure 5). In spring samples, we found filoplumes of a bird as well as hair and bone remains from a mammal, *Rattus rattus*. In two fecal samples from an adult female and an adult male, we found bone remains of juvenile *P. lilfordi*.

## 4. Discussion

On the Balearic Islands, there are as many as 46 different populations of the Balearic wall lizard, found on islands and islets of extremely variable size and ecological conditions. This situation has promoted comparative studies among populations, as well as syntheses of the ecological and natural history traits of this species [37]. However, this approach prevents the identification of the characteristics of each population, especially in the case of very isolated populations that are difficult to access and for which data are scarce or absent. There is not even basic information available on the morphometry of the lizards, their sexual dimorphism or their body condition. Colom [5] only mentions that lizards from Colomer had an SVL + tail of 130 mm, with a maximum value of 150 mm. The morphometric information given by Salvador [2] was also limited and only derived from a subsample of an unknown number of specimens deposited in the Natural History Museum of London (UK).

Sexual dimorphism is clear in lizards from Colomer, as in several populations of *P. lilfordi* [21]. From the morphological viewpoint, adult males of the Colomer population occupy an isolated position among all known populations of the Lilford’s Wall Lizard, only being close to some islets of the Cabrera Archipelago and Mel Islet (Menorca) [21]. Colomer’s lizards could be characterized as having a black color with cobalt blue spots arranged in two or three longitudinal series on each side of the ventral zone (Figure 4B). The most internal series have smaller blue spots. Many species also have cobalt blue spots on their flanks. According to the description of the subspecies *Podarcis lilfordi colomi*, these characteristics are similar in the population that inhabits the Imperial islet in the Cabrera Archipelago [3] and, in general, the remaining melanistic populations of Mallorca and the Cabrera islets [21]. Lizards from Colomer were not included in the analyses of dorsal pattern and coloration but, as a melanistic population, they could be ranged with other populations where black color dominates [21]. In adult females and juveniles, the back coloration can be full or with a reddish nuance and some longitudinal shaded stripes.

We found a significantly higher proportion of males than females with digit amputations. This result likely indicates intense intraspecific competition [38], because of the high population density. Unfortunately, we only have data derived from one sample from the summit area obtained in 2022, and the rest of the data on amputations come from museum specimens (NHML), for which there is no information on origin, although it is likely that they were captured in the slope zone.

In Colomer, we did not find any correlations between body size and ectoparasite load, as described in other insular populations of *Podarcis* spp. [23]. The 100% of prevalence of ectoparasites (mites) and blood parasites in Colomer is higher than the prevalence values recorded in other populations of *P. lilfordi* [23,39,40], but similar to values found in populations of the Cabrera Archipelago [41] and in other lacertid lizards, such as *Gallotia atlantica* [42]. Body condition was not correlated with ectoparasite load or blood parasite load. However, we found clear differences between slope and summit areas. Lizards from the slope showed a significantly higher ectoparasite load and lower blood parasitism, while at the summit, lizards showed an opposite trend, with higher blood parasitism and lower ectoparasite load. The higher intensity of blood parasites at the summit area can be related with the higher population density here [41]. A high population density would promote frequent intraspecific interactions and, particularly, aggressive male–male interactions. In turn, a notable physiological effect of these interactions is the increase in testosterone levels that produces an inmunodepressive effect and gives rise to a higher susceptibility to parasite infection [43,44]. This situation gives rise to a surprising negative correlation between external parasite load and blood parasite load in individual lizards. This result is difficult to interpret. For instance, Drechsler et al. [45] showed a negative relationship between mite abundance and *Lankestarella* spp. blood parasites in *Psammodromus algirus*, but an opposite result in *Acanthodactylus erythrurus*. This result could be an indication that vectors other than mites can infect lizards with blood parasites. In *Podarcis erhardii*, lizards with higher tick loads showed a higher probability of being infected with blood parasites (hemogregarines), while the number of mites does not have a significant effect on the number of hemogregarines [39]. Thus, the relationship of ectoparasitism and blood parasitism can be extremely complex and is probably influenced by several ecological and physiological factors. Only an experimental approach or a large comparative study could offer a deeper explanation of these preliminary findings.

Lizards from Colomer consumed an omnivorous diet, as in several populations of the Lilford’s Wall lizard [16,17], with the inclusion of remains from mammal and bird carcasses. We also found a couple of cases of cannibalism. As in most populations studied in Mallorca and Menorca, the diet of *P. lilfordi* is significantly less diverse in summer than in spring, due to the summer disappearance of some prey types and, above all, the intense consumption of pseudofruits of *Ephedra fragilis* in June. The joint pine is a pioneer evergreen gymnosperm shrub [46], a mast seeder with a locally synchronized production of pseudofruits in years of massive cone production [47]. In Colomer, the Lilford’s Wall lizard is the main seed disperser for *E. fragilis*. Thus, in masting years, as 2024, the consumption of pseudofruits of *E. fragilis* can be very important, as reported on Dragonera Island (Mallorca, Balearic Islands) [47,48].

The time series of the lizard density measurements on Es Colomer is still too short to analyze in relation with biotic or abiotic factors, or to establish trends [37]. The summit area is, apparently, optimal for lizards, which here reach densities greater than 4000 individuals per hectare. In fact, the density recorded in 2024 (Table 2) is the largest of all known densities of the Lilford’s Wall lizard [37]. The high density is characteristic of the very small coastal islets where *P. lilfordi* lives [9,37]. With this abundance in an island of more than three hectares, Colomer can be viewed as a population with a good conservation status. In addition, the behavior of Colomer lizards is typical of a population that has barely been visited, given the difficulty of access. These lizards are not afraid of humans and are remarkably trusting, compared to other populations of *P. lilfordi*, or to populations of other Mediterranean species present on small islets [49]. During the summer, the coastal area of Colomer is visited by several sports boats, but did not observe any attempts to disembark. On our first visits, we detected the presence of the ship rat *Rattus rattus* in the lower areas of the eastern slope of the islet. On the last two visits, in August 2022 and June 2024, we also detected the presence of ship rats even in the summit area. As in so many other populations, we did not detect any effect of the rats on the lizard population, and we therefore consider that no intervention should be made into the population for that reason (see [37] for a discussion of this conservation issue).

## 5. Conclusions

Colomer Island is home to a population of the Lilford’s Wall lizard that is unique within the known populations of the species on the Balearic Islands. This is not only because it is an extraordinary representation of the genetic variants that inhabited Mallorca Island before the Holocenic extinction, but also because it represents a unique case of adaptation to the extreme conditions of these small coastal islets. Colomer lizards show the highest population density known today amongst *P. lilfordi*, a marked sexual dimorphism, a high ectoparasitic and blood parasite load, and an omnivorous and opportunistic diet. This includes the frequent consumption of plant matter, with an intensive exploitation of specific plant species during masting periods, and the consumption of the remains of bird and mammal carcasses, as well as the sporadic capture of conspecifics. For all these reasons, the conservation of this independent and unrepeatable evolutionary history represented by Colomer lizards must be a priority, and our findings reinforce our proposal that each and every one of the current populations of Lilford’s Wall lizard constitute unique and non-interchangeable Evolutionarily Significant Units (ESU) that deserve strict protection [21].

## Figures and Tables

**Table 1:** Morphometry and scalation of *P. lilfordi* from Es Colomer Island. SVL: Snout–vent length. Tail: length of intact tail. Weight (in grams). PL: pileus length. HH: Head height. HW: Head width. HLL: Hind leg length. LAM: Lamellae. FEM: Femoralia. GUL: Gularia. DOR: Dorsalia. VENT: Ventralia. COLL: Collaria. All body measurements in mm. We give the mean ± standard error (SE) for adult males and females, F-value and degrees of freedom (d.f.) of the ANOVA or ANCOVA analyses and their corresponding *p*-values. In the last two columns we show the F-values and *p*-values of the interaction of sex and SVL (see more details in the text).

Trait	Males Mean ± SE (n)	Females Mean ± SE (n)	F-Value	d.f.	*p*-Value	F-Value of Interaction	*p*-Value of Interaction
SVL	74.32 ± 0.53 (48)	66.86 ± 0.89 (28)	59.85	1.74	4.08 × 10^−11^		
Tail	130.75 ± 2.18 (8)	116.21 ± 4.63 (7)	10.76	3.11	0.007	1.67	0.22
Weigth	11.62 ± 0.31 (23)	6.84 ± 0.42 (21)	141.58	3.39	1.49 × 10^−14^	1.44	0.24
PL	18.04 ± 0.14 (46)	15.31 ± 0.19 (21)	180.30	3. 62	2.2 × 10^−16^	1.16	0.28
HH	8.74 ± 0.11 (47)	7.06 ± 0.1 (21)	92.10	3.63	6.09 × 10^−14^	0.09	0.77
HW	8.3 ± 0.07 (46)	7.29 ± 0.25 (21)	39.86	3.62	3.26 × 10^−8^	0.34	0.56
HLL	39.43 ± 0.28 (42)	34.47 ± 0.5 (20)	89.36	3.57	2.83 × 10^−13^	0.85	0.36
LAM	30.88 ± 0.42 (34)	30.67 ± 0.4 (12)	0.04	3.42	0.84	0.20	0.66
FEM	22.92 ± 0.4 (38)	22.36 ± 0.75 (11)	0.37	3.44	0.55	0.27	0.60
GUL	35.72 ± 0.77 (25)	32.83 ± 1.25 (6)	2.83	3.27	0.10	0.57	0.45
DOR	93.23 ± 1.62 (26)	84.83 ± 1.4 (6)	7.51	3.27	0.01	0.0001	0.99
VENT	23.96 ± 0.23 (26)	26 ± 0.26 (6)	14.93	3.27	0.0006	0.46	0.50
COLL	11.68 ± 0.23 (25)	10.67 ± 0.61 (6)	3.95	3.26	0.057	5.46	0.02

**Table 2:** Lizard densities at Colomer in May 2008, August 2022, and June 2024. For each year, we give the total number of lizard contacts during the line transects (N), the length (in m) of the transects, the log-density ± standard error (SE), the z-value of the Wald statistics and its corresponding *p*-value, the Akaike information value (AIC), the density ± SE (individuals/hectare) and the detectability function, g(x) (see more details in the text).

Date	N	l (m)	Density (log) ± SE	z	*p*	AIC	Density (ind./ha) ± SE	g(x)
2008	54	140	8.3 ± 0.169	49.2	0	130.06	4007 ± 675	0.5129
2022	19	79.3	7.45 ± 0.288	25.9	1.11 × 10^−147^	24.07	1723 ± 496	0.3476
2024	113	108.5	8.56 ± 0.12	71.4	0	225.31	5195 ± 623	0.5012

**Table 3:** Diet of *P. lilfordi* in Colomer Island. Frequency, n—prey frequency of each taxon, n%—percentage of prey of each taxon in relation to prey number. Presence, np—number of scats in which each taxon is present and %p—percentage of scats in which each taxon is present. The number of prey items from a given taxon is estimated from the count of significant elements (see more details in the text).

Taxa	Overall Diet				Spring Diet				Summer Diet			
	Frequency		Presence		Frequency		Presence		Frequency		Presence	
	n	n%	np	%p	n	n%	np	%p	n	n%	np	%p
Gastropoda	26	3.7735	26	12.50	7	2.7777	7	6.1946	19	4.3478	19	20.000
Pseudoscorpionida	2	0.2902	2	0.9615	1	0.3968	1	0.8849	1	0.2288	1	1.0526
Araneae	4	0.5805	4	1.9230	3	1.1904	3	2.6548	1	0.2288	1	1.0526
Acari	1	0.1451	1	0.4807	0	0	0	0	1	0.2288	1	1.0526
Isopoda	43	6.2409	42	20.1923	30	11.9047	29	25.6637	13	2.9748	13	13.6842
Dictyoptera	1	0.1451	1	0.4807	1	0.3968	1	0.8849	0	0	0	0
Isoptera	5	0.7256	5	2.4038	1	0.3968	1	0.8849	4	0.9153	4	4.2105
Homoptera	6	0.8708	6	2.8846	4	1.5873	4	3.5398	2	0.4576	2	2.1052
Heteroptera	9	1.3062	9	4.3269	7	2.7777	7	6.1946	2	0.4576	2	2.1052
Diptera	3	0.4354	3	1.4423	3	1.1904	3	2.6548	0	0	0	0
Lepidoptera	8	1.1611	7	3.3653	8	3.1746	7	6.1946	0	0	0	0
Coleoptera	68	9.8693	53	25.4807	35	13.8888	29	25.6637	33	7.5514	24	25.2631
Hymenoptera	12	1.7416	10	4.8076	8	3.1746	6	5.3097	4	0.9153	4	4.2105
Formicidae	391	56.7489	125	60.0961	126	50.0000	65	57.5221	265	60.6407	60	63.1578
Arthropoda undet.	9	1.3062	9	4.3269	8	3.1746	8	7.0796	1	0.2288	1	1.0526
Larvae	13	1.8867	13	6.2500	6	2.3809	6	5.3097	7	1.6018	7	7.3684
Birds	1	0.1451	1	0.4807	1	0.3968	1	0.8849	0	0	0	0
Lizards	2	0.2902	2	0.9615	2	0.7936	2	1.7699	0	0	0	0
Mammals	1	0.1451	1	0.4807	1	0.3968	1	0.8849	0	0	0	0
Seeds	84	12.1915	38	18.2692	0	0	0	0	84	19.2219	38	40.00
Total	689	100			252	100			437	100		

## Data Availability

The data presented in this study are available on request from the corresponding author.

## Abbreviations

The following abbreviations are used in this manuscript:

ESU	Evolutionarily Significant Unit
